# Prevention of delirium (POD) for older people in hospital: study protocol for a randomised controlled feasibility trial

**DOI:** 10.1186/s13063-015-0847-2

**Published:** 2015-08-08

**Authors:** John Young, Francine Cheater, Michelle Collinson, Marie Fletcher, Anne Forster, Mary Godfrey, John Green, Shamaila Anwar, Suzanne Hartley, Claire Hulme, Sharon K Inouye, David Meads, Gillian Santorelli, Najma Siddiqi, Jane Smith, Elizabeth Teale, Amanda J Farrin

**Affiliations:** Academic Unit of Elderly Care and Rehabilitation, University of Leeds, Bradford Institute for Health Research, Temple Bank House, Bradford Royal Infirmary, Duckworth Lane, Bradford, BD9 6RJ UK; School of Nursing Sciences, Faculty of Medicine and Health Sciences, University of East Anglia, Edith Cavell Building, Norwich Research Park, Norwich, NR4 7TJ UK; Clinical Trials Research Unit, Leeds Institute of Clinical Trials Research, University of Leeds, Leeds, LS2 9JT UK; Leeds Institute of Health Sciences, Charles Thackrah Building, 101 Clarendon Road, Leeds, UK; Academic Unit of Health Economics, Leeds Institute of Health Science, University of Leeds, Leeds, LS2 9JT UK; Department of Medicine, Beth Israel Deaconess Medical Center, Harvard Medical School, Boston, MA 02215 USA; Aging Brain Center, Institute for Aging Research, Hebrew SeniorLife, 1200 Centre Street, Boston, MA 02131 USA; Bradford District NHS Care Trust New Mill, Victoria Road, Saltaire, BD18 3LD UK

**Keywords:** Delirium, Elderly, Feasibility study, Hospital, Process of care

## Abstract

**Background:**

Delirium is the most frequent complication among older people following hospitalisation. Delirium may be prevented in about one-third of patients using a multicomponent intervention. However, in the United Kingdom, the National Health Service has no routine delirium prevention care systems. We have developed the Prevention of Delirium Programme, a multicomponent delirium prevention intervention and implementation process. We have successfully carried out a pilot study to test the feasibility and acceptability of implementation of the programme. We are now undertaking preliminary testing of the programme.

**Methods/Design:**

The Prevention of Delirium Study is a multicentre, cluster randomised feasibility study designed to explore the potential effectiveness and cost-effectiveness of the Prevention of Delirium Programme. Sixteen elderly care medicine and orthopaedic/trauma wards in eight National Health Service acute hospitals will be randomised to receive the Prevention of Delirium Programme or usual care. Patients will be eligible for the trial if they have been admitted to a participating ward and are aged 65 years or over. The primary objectives of the study are to provide a preliminary estimate of the effectiveness of the Prevention of Delirium Programme as measured by the incidence of new onset delirium, assess the variability of the incidence of new-onset delirium, estimate the intracluster correlation coefficient and likely cluster size, assess barriers to the delivery of the Prevention of Delirium Programme system of care, assess compliance with the Prevention of Delirium Programme system of care, estimate recruitment and follow-up rates, assess the degree of contamination due to between-ward staff movements, and investigate differences in financial costs and benefits between the Prevention of Delirium Programme system of care and standard practice. Secondary objectives are to investigate differences in the number, severity and length of delirium episodes (including persistent delirium); length of stay in hospital; in-hospital mortality; destination at discharge; health-related quality of life and health resource use; physical and social independence; anxiety and depression; and patient experience.

**Discussion:**

This feasibility study will be used to gather data to inform the design of a future definitive randomised controlled trial.

**Trial registration:**

ISRCTN01187372. Registered 13 March 2014.

## Background

Delirium (also called *acute confusion* or *toxic confusion*) is a common condition affecting older people, especially those who have dementia. In the United Kingdom, national policy has identified the improved management of delirium as a priority [1], and national guidelines are available [2, 3]. Yet, there is much evidence that delirium represents a largely unrecognised condition affecting older people within health care services internationally [4]. It is also now recognised that health care systems and services frequently have attributes that unintentionally stimulate or aggravate delirium in older people [4]. Demographic transitions of populations have required health services internationally to address the needs of older people as a priority. The National Health Service (NHS) in the United Kingdom has been slow to recognise that the modern general hospital is increasingly an older person’s facility, with over two-thirds of the beds occupied by people older than 65 years of age, many of whom have complex needs requiring multidisciplinary care [5]. General hospitals, however, have poorly developed care systems that are not yet fully aligned to the needs of this vulnerable group [6].

Delirium is the most frequent complication of hospitalisation for older people [7], with an occurrence in general medical and old age medicine wards of 29–64 % [8]. The large majority of delirium episodes remain undetected or misdiagnosed by ward teams [3]. Delirium is an unpleasant experience for patients [9]. The experience of delusions and hallucinations during delirium may be associated with the development of later neuropsychiatric symptoms [10]. The development of delirium is associated with increased mortality rates, functional decline, falls and increased requirement for institutional care [8]. Symptoms of delirium can persist in some patients for up to 12 months [11], and in-hospital delirium has also been linked with depression after hospital discharge [10].

*Persistent delirium* refers to the situation in which the delirium symptoms do not resolve [12]. Dementia is the strongest risk factor for persistent delirium [13–16], but age and comorbidity are additional factors [17]. The authors of a systematic review of 18 studies involving 1322 hospital patients older than 50 years of age (mean age, 72–89 years; median age, 82 years) with prevalent or incident delirium reported the rates of persistent delirium as 45 %, 33 %, 26 % and 21 % at hospital discharge and 1, 3 and 6 months postdischarge, respectively [12]. Persistent delirium is directly linked to poor outcomes: Each 48 hours spent with delirium is associated with an 11 % increase in mortality [18].

Perhaps the most important aspect of delirium is that multicomponent non-pharmacological interventions can significantly reduce delirium incidence (odds ratio, 0.47; 95 % confidence interval, 0.38–0.58) [19]. Intervention delivery strategies have included additional staff and volunteers [20–22], proactive geriatric consultation [23], training family members [24] and sustained education [25, 26]. Not all studies have found a reduction in delirium incidence [27], and a high degree of protocol adherence is a critical issue for success [28]. Researchers have primarily examined outcomes in a research context and, with few exceptions, have not addressed how to achieve sustainability in routine care [29]. The main exception has been the Hospital Elder Life Program (HELP), which has been widely sustained in routine care outside the United Kingdom [30–32].

However, the existing delirium evidence base is sufficiently robust to present a clear opportunity for the NHS to address the necessary professional skills, cultural aspects and service design in such a way as to prevent or attenuate delirium in older people [11]. There are strong arguments based on international research to suggest that delirium prevention and its improved management should be fundamentally predicated within high-quality care processes for older people [33–37], particularly care processes that recognise the specific needs of older people with cognitive impairment. Clinical teams need validated support systems to help them achieve this [38]. Importantly, there is evidence to indicate that multicomponent interventions can improve outcomes [11, 19]. Unfortunately, the NHS does not have routine care systems capable of minimising the impact of this common condition. Consequently, many older patients are currently disadvantaged in terms of outcomes, and considerable additional acute bed days are unnecessarily used [6].

A multicomponent intervention to prevent delirium was developed as part of a programme of interlinked studies with the aim of improving delirium prevention for older people in hospital and reduce the burden of delirium for the NHS. The development work followed the Medical Research Council framework for complex interventions [39]. First, we convened a development team (consisting of a senior manager, clinicians, matron, nurse consultant, specialist nurse, staff nurse, therapist, manager of the volunteer service, patient representative, care assistant and ward clerk) to review an evidence-based, multicomponent delirium prevention system of care (HELP) previously developed in the United States [20, 21]. This was augmented by up-to-date evidence for delirium prevention from the National Institute for Health and Care Excellence (NICE) [3]. In this review process, we identified candidate implementation and delivery strategies to produce the Prevention of Delirium Programme (POD) system of care. Second, we performed an implementation pilot in five wards in four NHS hospitals to test the feasibility and acceptability of the POD system of care. Key findings emerged about implementation and delivery, and some modifications of the content and presentation of the POD manuals and associated toolkit were made before implementation in the feasibility study [40]. Here we report the protocol for a third study designed to explore preliminary estimates of effectiveness and cost-effectiveness of the modified version of the POD system of care in older patients at risk of developing delirium. The data obtained from this feasibility study will be used to inform the design of a possible future definitive randomised controlled trial (RCT).

## Methods/Design

### Aims and objectives

We aim to conduct a pragmatic, multicentre, cluster randomised, controlled feasibility study to explore the potential effectiveness and cost-effectiveness of the POD system of care versus standard care practice in older patients at risk of developing delirium who are admitted to hospital for emergency care. The study objectives relate to gathering data to inform the feasibility of conducting a definitive RCT. The primary objectives are to

1. Provide a preliminary estimate of the effectiveness of POD compared with standard care as measured by the incidence of new-onset delirium within 10 days of hospital admission (anticipated primary outcome for a definitive trial)
2. Assess the variability of the incidence of new-onset delirium within 10 days of hospital admission in both treatment groups
3. Estimate the intracluster correlation coefficient and likely cluster size
4. Assess barriers to the delivery of the POD system of care
5. Assess compliance with the POD system of care
6. Estimate recruitment and follow-up rates at both patient and cluster levels
7. Assess the degree of contamination at ward level due to between-ward staff movements
8. Investigate differences in financial costs and benefits between the POD system of care and standard practice

Our secondary objectives are to investigate differences in the number, severity and length of delirium episodes (including persistent delirium); length of stay in hospital; in-hospital mortality; destination at discharge; health-related quality of life and health resource use; physical and social independence; anxiety and depression; and patient experience.

### Setting

#### Ward inclusion criteria

The study will be conducted in eight NHS hospitals (16 wards) which care for those at risk of developing delirium (geriatric medicine or orthopaedic trauma wards). The following ward criteria were identified in the preliminary study and were applied to maximise the likelihood of successful implementation of the POD system of care:

1. Involvement of senior nurse, ward manager and voluntary services manager (if voluntary services were selected by local staff as a component of the intervention)
2. Named person responsible for supporting the implementation of POD (e.g., a senior nurse)
3. Dedicated time (equivalent to 1 day per week) of an experienced senior nurse to lead the implementation period (funded with the research grant)
4. Adequate staffing levels as assessed in reference to guidance on nurse staffing [41, 42]

#### Ward exclusion criteria

Wards that previously participated in research leading to the development of the POD system of care, as well as wards that intend to implement a delirium prevention initiative during the duration of the study, will not be eligible, because these sites might have enhanced skills or knowledge about delirium prevention.

### Patient inclusion criteria

Patients aged 65 years and older who are admitted to a participating ward will be eligible for study participation.

### Patient exclusion criteria

Patients meeting any of the following criteria will not be eligible for study entry:

- Patients with prevalent delirium (diagnosed by using the confusion assessment method (CAM) [43]
- Patients with a discharge from hospital planned within 48 hours of admission
- Patients for whom a delirium assessment (via the CAM) has not been performed within 24 hours of admission to the ward (for elderly care patients) or for whom a delirium assessment has not been performed preoperatively (e.g., patients with hip fracture)
- Patients who have not provided consent or, for patients who lack the capacity to provide consent, where there is no agreement from a consultee for trial participation within 48 hours of the patient’s admission to the ward
- Patients receiving end-of-life care (because it is unlikely follow-up data will be collected from these patients)
- Patients from another ward who are not under the care of the ward medical team

### Unit of randomisation

**Fig. 1:**
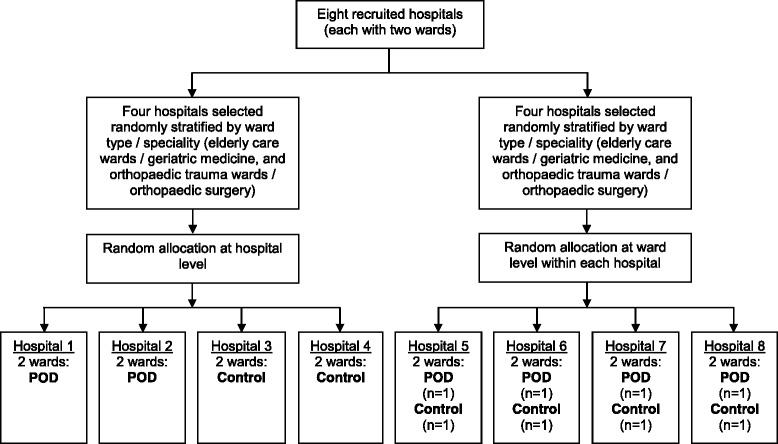
Randomisaion overview

Cluster randomisation has been chosen to reduce between-group contamination, as the POD system of care is a ward-based intervention which aims to affect staff skills, knowledge and clinical practice. As patients on a ward receive the allocated treatment, it is not possible to use the patient as the unit of randomisation. It is not known whether treatment contamination might occur between wards owing to staff movement, and therefore this will be investigated by randomising half of the hospitals at the hospital level (control or intervention) and half of the hospitals at the ward level. Randomisation will stratify by ward type (geriatric medicine and orthopaedic trauma) and is a two-stage process which will be performed centrally by the statistician at the Clinical Trials Research Unit (CTRU). Sites will first be randomised 1:1 between site- (i.e., hospital)-level allocation (both wards in the hospital receive the same intervention) and ward-level allocation (each ward in the hospital receives a different intervention). Those sites selected for site-level allocation will then be further randomised 1:1 for both of their wards to receive either POD or control. Wards in those sites selected for ward-level allocation will be randomised 1:1 to receive either POD or control (Fig. 1).

### Training in study procedures

Research assistants (RAs) will be appointed at each hospital to recruit study participants and perform study procedures.

Training in the use of the CAM [43] to detect delirium will be provided by the central trial team in consultation with the instrument developer. The diagnostic version of the CAM has four items: (1) acute onset and fluctuating course, (2) inattention, (3) disorganised thinking and (4) altered consciousness level. A diagnosis of delirium requires positive results for items (1) and (2) and either (3) or (4). Administration of the CAM takes 3–5 minutes and is informed by a formal cognitive assessment. In validation studies, researchers have reported a sensitivity range of 94–100 % and a specificity range of 90–95 % [44].

The RAs, before recruitment, will follow the recommended training procedure [45] by co-assessing up to 10 patients with or without delirium to ensure acceptable inter-rater reliability (IRR). This prerecruitment training method will also provide an important opportunity for the RAs to work alongside the ward staff and become more integrated and familiar with ward routines and practices. The RAs will use a standard procedure to undertake the CAM [45]. First, where possible, relevant information will be obtained from ward staff and/or from patients’ relatives and/or carers. Then, following an informal general conversation with the patient, formal cognitive testing will be undertaken using the Abbreviated Mental Test Score [46] and the calendar months backwards test [47]. Finally, the CAM questions will be scored (Table 1). Ongoing sessions to discuss coding questions will enhance reliability.

**Table 1:** Confusion assessment method questions and source of information

Question	Source of information
1. Acute onset and fluctuating course	Ward staff or relative/carer who knows the patient’s baseline mental status and has observed the patient over time
	Medical/nursing notes, including the baseline Abbreviated Mental Test Score
2. Inattention	Informal general conversation
	Formal cognitive testing: Abbreviated Mental Test Score [46], months of the year backwards test [47]
3. Disorganised thinking	Informal general conversation
	Formal cognitive testing: Abbreviated Mental Test Score [46], months of the year backwards test [47]
4. Altered level of consciousness	Ward staff
	Informal general conversation
	Observation

Confusion Assessment Method. © 1988, 2003, Hospital Elder Life Program. Used with permission

The RAs will undertake further IRR assessments approximately halfway through the data collection period.

### Intervention

POD is a manualised, multicomponent intervention and systematic implementation process designed to secure ward practice changes, potentially enhanced by the involvement of hospital volunteers. It builds on current best evidence on delirium prevention [3], conceptually driven empirical research [48] and reviews of implementation [29, 49, 50].

The POD intervention comprises actions centred on 10 targeted risk factors associated with the development of delirium among those who are vulnerable on of the basis of predisposing risk [3]. These interventions directly affect the patient experience of care and include optimising hydration and nutrition, reducing environmental threats (excessive noise, multiple moves), increasing orientation to time and place, improving communicative practices (personally meaningful interaction and cognitive stimulation), supporting and/or encouraging mobility and better management of pain and infection. The implementation process is supported and reinforced through raising awareness and training of staff and volunteers in delirium prevention. Alongside this, staff will be involved in an action-planning cycle of observation and audit of current practice to establish what needs to be put in place to introduce the POD system of care. The principles underpinning POD and the steps in the change process to facilitate action on the intervention are standard and generalisable, although the specific systems or mechanisms are flexible, depending on preexisting practice and local decision-making.

Wards randomised to the POD system of care will undergo a 6-month implementation period to allow the intervention to be embedded into ward practice before patient recruitment. Local implementation teams, including a study-specific ward nurse, will be convened, and training will be provided in the implementation and delivery of the POD system of care by the central trial team. Progress on implementation will be monitored by the central trial team through regular site visits and telephone and/or email contact and will be tracked through completion of an internal milestone checklist embedded within the POD manual.

### Usual-care control group

Wards randomised to the usual-care control group will continue to deliver care as determined by local policies and practices.

### Recruitment and consent

Following a 6-month implementation period for the POD intervention, study participants will be recruited over a 6-month period. All patients 65 years of age or older admitted to a participating ward will be considered for enrolment into the study in a three-stage recruitment process:

1. Initial screening for eligibility will be established through a liaison between the RA and the ward staff (stage 1).
2. Patients will be screened for the presence of delirium by the RA within 24 hours of admission to the ward (e.g., elderly care patients) or preoperatively (e.g., hip fracture patients) (stage 2).
3. Patients who do not have prevalent delirium will then be invited by the RA to participate in the trial within 48 hours of admission to the ward. The RA will obtain informed consent from the participant or, if the patient lacks the capacity to provide consent, from a personal or nominated consultee (stage 3).

**Fig. 2:**
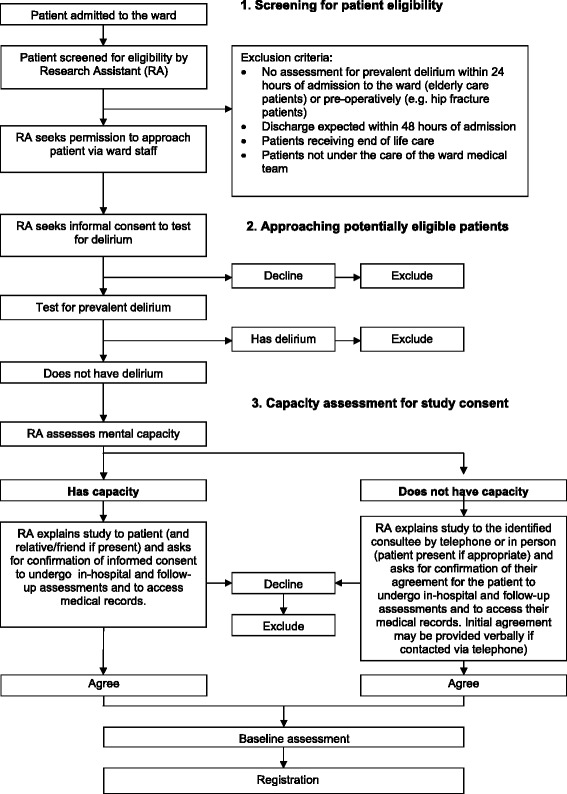
Recruitment overview

Figure 2 provides an overview of the recruitment process.

### Recruitment of patients at risk of reduced capacity

Capacity is likely to be a major issue in this study population, as the presence of dementia may reduce patients’ capacity to give consent.

The Mental Capacity Act 2005 (MCA; http://www.legislation.gov.uk/ukpga/2005/9/contents) requires that those lacking capacity are only included in research that is likely to be of direct benefit to those taking part or to benefit the particular population under study. In this study, ward patients receiving the POD intervention may benefit directly from improved quality of care. Another potential direct benefit for those taking part in the study is that screening assessments for delirium on admission to the ward may identify people with delirium at an earlier stage, allowing earlier treatment.

Excluding those without capacity from this research would not be ethical, as it would compromise the generalisability of findings by recruitment of an unrepresentative study sample and would exclude this vulnerable group from the benefits of research evidence in improving practice.

To comply with the MCA, ethical approval was obtained to seek personal consultee or nominated consultee agreement for patients with impaired capacity and for participants who lost capacity following the provision of informed consent.

### Data collection

The screening data will be obtained by the RA in consultation with the attending ward staff and will include demographic characteristics, admission details and, where applicable, assessment of capacity and a delirium screen and/or CAM severity rating (CAM-S) [43, 45] for all patients older than 65 years of age admitted to the ward. Baseline assessment will be conducted by the RA and will include admission details, reason for admission, medical history (including the Charlson comorbidity index) [51], existing hearing and or visual impairments, current medication use, illness severity (using an early warning score [National Early Warning Score or equivalent]), CAM-S), cognitive assessment (history of dementia and Abbreviated Mental Test Score) [46], medications and living arrangements. Participants will complete questionnaires relating to physical and social independence (Nottingham Extended Activities of Daily Living Scale) [52] and health-related quality of life (EuroQol [EQ-5D]) [53]. The RA will perform daily cognitive and CAM assessments for up to 10 days postadmission (or until discharge, whichever is sooner) to detect the incidence of new delirium.

At 30 days after admission to the ward, the RA will perform a cognitive assessment and CAM and ask the patient to complete questionnaires about health-related quality of life (EQ-5D) [53], anxiety and depression (using the Clinical Anxiety Scale [54] and the Geriatric Depression Scale Short Form [55], respectively) and about patient experience using selected questions in the patient-reported experience measure from the National Audit of Intermediate Care [56]. At the point of discharge, the RAs will collect data on the date of discharge (or date of death), causes of death (if known), falls occurring (and reason for fall if known) between the date of consent and date of discharge using local recording procedures (e.g., falls incidence records) and discharge destination.

At 3 months after admission to the ward, postal questionnaires will be used to provide information on physical and social independence (Nottingham Extended Activities of Daily Living Scale) [52], health-related quality of life (EQ-5D) [53], health care resource use (post discharge contact with health and social care services) and information on living arrangements. Proxy completion of the questionnaires and the resource use will be permitted.

A summary of the assessments to be used is given in Table 2.

**Table 2:** Summary and timing of assessments

Assessment	Screening/recruitment	Baseline	Daily (up to 10 days from date of admission)	30 days (postadmission)	Discharge	Follow-up (3 mo postadmission)
Demographic data (age, sex, ethnicity)	X					
Admission details (date and time of admission to hospital and ward)	X					
Assessment of capacity	X					
Delirium screen (confusion assessment method) and delirium severity rating (CAM-S)	X		X	X		
Date of discharge and discharge destination (living alone, living with another person, nursing home, residential care home or other)					X	
Reason for admission (hip fracture, other orthopaedic condition, medical condition) and medical history (Charlson comorbidity index, existing hearing and/or visual impairments, current medication use [benzodiazepines, opiates, H_1_ antihistamines])		X				
Cognitive impairment: (1) history of dementia, (2) low Abbreviated Mental Test Score on admission		X				
Illness severity using National Early Warning Score or equivalent		X				
Living arrangements (living alone, living with another person, nursing home, residential care home or other)		X			X	X
Nottingham Extended Activities of Daily Living Scale^a^		X				X
EuroQol EQ-5D^a^		X		X		X
Geriatric Depression Scale Short Form^a^				X		
Clinical Anxiety Scale				X		
Patient-reported experience measure				X		
Falls (and reason for fall if known)	Occurring between the date of consent and date of discharge					
Health care resource use (postdischarge contact with health and social care services)^a^						X

*Abbreviation: CAM-S* confusion assessment method severity rating

^a^Proxy completion permitted where appropriate

Compliance with the POD system of care will be assessed by the central trial team through review of site-specific POD documentation, case note review and researcher-conducted observations of ward care using the method employed in the 2013 National Audit of Dementia Care in General Hospitals [57]. Sustainability will be assessed through observations of ward care approximately 3–6 months post recruitment through central trial team researcher-conducted observations of ward care. Information will be collected on intervention delivery, recruitment uptake, follow-up rates, missing data, length of hospital stay, staff movement (to allow for an assessment of potential contamination), the number of falls between consent and discharge, and deaths occurring up to 3 months post admission. We will also collect related and unexpected serious adverse events.

### Sample size

We aim to recruit 720 patients into this feasibility study. This is based on the following assumptions:

- Average length of stay of 14 days and 25-bed wards will provide a recruitment pool of 4800 patients over the course of 6 months.
- Fifty percent of patients are assumed to be at risk of delirium, giving 2400 eligible patients.
- On the basis of previous studies of comparable populations [58, 59], 30 % of patients will consent.

Because this is a feasibility study, a formal power calculation is not appropriate, as effectiveness is not being evaluated. The results generated from this study will be used to inform the power calculation for a possible definitive study.

### Analysis

Statistical analysis is the responsibility of the University of Leeds CTRU statistician, and a final statistical analysis plan will be written before any analysis is undertaken. All analyses and data summaries will be conducted on the intention-to-treat (ITT) population, defined as all participants registered, regardless of non-compliance with the protocol or withdrawal from the study. No formal interim analyses are planned, and final analysis will take place when all available data have been received. The analysis will be focused on descriptive statistics and confidence interval estimation rather than on formal hypothesis testing.

#### Primary analysis

##### Estimation of effectiveness for a possible definitive randomised controlled trial

To inform the sample size calculation for a possible definitive trial, we will calculate the incidence of new-onset delirium within 10 days of admission by ward type, overall and per arm, together with corresponding 95 % confidence intervals. Estimation of effectiveness will be carried out on the ITT population using multilevel logistic regression adjusting for NICE risk factors for delirium, medications which could impact on the development of delirium, hearing impairment and use of hearing aid, visual impairment and ward type. Ward type will be fitted as a random effect in the model. Odds ratios, 95 % confidence intervals and *p* values will be reported. The number of new patients admitted per ward during the recruitment period will be used to estimate cluster size. The intracluster correlation coefficient will be calculated using the incidence of new-onset delirium expressed as a proportion.

##### Assessment of intervention delivery and compliance

The progress of wards randomised to POD will be assessed by completion of the internal milestone checklist, which includes the length of time taken to complete each core task (staff education, review of current practice and ward systems) and overall time spent implementing POD, the number of sites and/or wards failing to progress through implementation milestones and reasons for failure, the number of sites and/or wards withdrawing during the implementation and delivery phases, the number of sites withdrawing from the study, timing and reason for withdrawal, case notes review and review of ward observation of care.

##### Estimation of recruitment uptake

To assess the feasibility of recruiting participants, the numbers of patients who are screened, are eligible, are assessed for delirium, have prevalent delirium, have capacity to consent, consent to trial registration and are registered will be summarised. Loss to follow-up at 30 days and 3 months, as well as the number, timing and reasons for withdrawals, will also be reported.

##### Assessment of the degree of contamination

The number of staff moving between study wards within sites will be reported. The incidence rates of new-onset delirium at the sites that were randomised at the hospital level will be calculated and compared with those from sites that were randomised at the ward level to assess the degree of contamination between wards.

#### Secondary outcomes

The following secondary outcomes will reported: (1) the number and length of delirium episodes during hospital stay and the number of days between admission to ward and the occurrence of delirium and (2) the outcome of the patient-reported questionnaires.

The number of deaths, the number of eligibility violations and the number of participants who fall during their hospital stays will also be reported overall and by arm. The type of living accommodation at 3 months will be compared with that at baseline and intended discharge location.

#### Cost-effectiveness

The primary analysis will take the perspective of the service provider, including the costs of health and personal social services. Discounting will not be applied, given the duration of the trial. Multilevel modelling and regression analysis (dependent variable: net benefit) will be the focus, which will allow us to control for baseline covariates and subgroup analysis [60]. Sensitivity analyses will be conducted to explore the impacts on the study results of using proxy versus patient reports, of using different methods to handle missing data and of using different volunteer time valuation techniques. In the secondary analysis, we will adopt a societal perspective, taking account of productivity costs and out-of-pocket expenditures incurred by patients, carers and volunteers.

### Criteria for continuation to the definitive randomised controlled trial

Guidelines for progression to a definitive RCT have been defined as a minimum of six wards (75 %) completing the milestone checklist (to provide assurance that the POD implementation was successful and the overall recruitment rate of at least 10 % of the total recruitment pool).

### Data monitoring

Trial supervision includes a core project team, a trial management group and a trial steering committee. For a feasibility study of this nature and duration, a separate data monitoring and ethics committee is not required; rather, the trial steering committee adopts a safety monitoring role, with the constitution of a subcommittee to review safety issues if this becomes necessary.

Data will be monitored for quality and completeness by the CTRU. Missing data (except individual items collected via postal questionnaires) will be chased until received, confirmed as not available or the trial is at analysis.

### Trial organisation and administration

The POD study is funded by the National Institute for Health Research under its Programme Grants for Applied Research Programme (grant RP-PG-0108-10037). The trial is sponsored by the Bradford Teaching Hospitals NHS Foundation Trust and is co-ordinated by the Academic Unit of Elderly Care and Rehabilitation (Bradford Teaching Hospitals NHS Foundation Trust and University of Leeds) and the CTRU (Leeds Institute of Clinical Trials Research, University of Leeds). The trial management group consists of the co-applicants and the teams from the co-ordinating units.

The study is adopted by the U.K. Clinical Research Network (CRN) and is supported in part by the CRN trained research staff. The trial is registered (ISRCTN01187372). The trial will be conducted in accordance with the principles of Good Clinical Practice, the NHS Research Governance Framework and through adherence to CTRU standard operating procedures. Ethical approval has been obtained through the U.K. National Research Ethics Service (13/YH/0400). The results of the study will be published in peer-reviewed publications and will be presented at relevant national and international conferences. We will work with the public and patient involvement representatives to develop lay reports to disseminate research findings to patient groups and the clinical teams at participating sites.

## Discussion

Delirium is a common condition and is the most frequent complication of hospitalisation in older people. It is strongly associated with adverse outcomes, and the burden to the patient, family, carer and the health care system is considerable. Evidence suggests that delirium can be prevented in more than one-third of patients through modifications to selected risk factors which can be incorporated and delivered within high-quality care processes for elderly patients. NICE demonstrated that such care systems are likely to be cost-effective [61] and recommended they should be widely deployed in routine care [62]. Our work comprises a sequence of linked studies to develop and evaluate a multicomponent intervention to prevent delirium. The protocol for the ongoing feasibility randomised trial described here has presented some critical implementation challenges, which are described below.

### Implementation of the POD system of care

A key aspect of this study is to implement and sustain a multicomponent system of care across different wards and hospitals. Earlier studies within this programme sought to identify the feasibility and acceptability of the POD system of care and to refine its content before commencing this feasibility study. This also provided insights into the conditions required to inform the site selection processes.

### Recruitment and retention

This is a cluster randomised controlled trial, and all participants admitted to a participating ward will receive the POD system of care or usual care. Consent for participation is therefore for the provision of data only. Nonetheless, we anticipate challenges in the identification, recruitment and retention of study participants in a population at risk of fluctuating capacity. We aim to minimise the risk of this through involvement of patient groups in the development of the participant information and recruitment processes and the provision of ongoing training and support to the RAs, obtaining ethical approval to obtain personal or nominated consultee agreement and implementing strategies to maximise data completion (e.g., allowing proxy completion and telephone reminders).

### Reliable assessment of delirium

CAM is widely used in the clinical research setting to reliably detect delirium and can be used by clinicians without psychiatric training. We propose to implement a prerecruitment training programme to ensure that the RAs are competent and reliable in conducting the CAM assessment, using validated procedures provided by the instrument developer, who is a member of the study team. We will also provide appropriate training manuals and further assess the IRR during the proposed research.

The findings from the POD study will inform the design and development of a future definitive trial, which could change the way in which care is provided to elderly patients in U.K. hospitals.

## Trial status

Recruitment completed on 28 February 2015 and the last 3 month follow-up took place on 19 June 2015. Analysis is underway and the trial results will be available in late 2015.

## Abbreviations

CAM: Confusion assessment method
CAM-S: Confusion assessment method severity rating
CRN: Clinical Research Network
CTRU: Clinical Trials Research Unit
HELP: Hospital Elder Life Program
IRR: Inter-rater reliability
ITT: Intention-to-treat
MCA: Mental Capacity Act
NHS: National Health Service
NICE: National Institute for Health and Care Excellence
POD: Prevention of Delirium Programme
RA: Research assistant
RCT: Randomised controlled trial
